# Editorial: Dynamics and Modulation of Synaptic Transmission in the Mammalian CNS

**DOI:** 10.3389/fnsyn.2019.00011

**Published:** 2019-04-09

**Authors:** Maria Elisa Calcagnotto, Alberto A. Rasia-Filho, Idan Segev

**Affiliations:** ^1^Neurophysiology and Neurochemistry of Neuronal Excitability and Synaptic Plasticity Laboratory, Department of Biochemistry, ICBS, Universidade Federal do Rio Grande do Sul, Porto Alegre, Brazil; ^2^Graduate Program in Biological Sciences: Biochemistry, Universidade Federal do Rio Grande do Sul, Porto Alegre, Brazil; ^3^Graduate Program in Neuroscience, Universidade Federal do Rio Grande do Sul, Porto Alegre, Brazil; ^4^Department of Basic Sciences/Physiology, Federal University of Health Sciences, Porto Alegre, Brazil; ^5^Department of Neurobiology, Edmond and Lily Safra Center for Brain Sciences, The Hebrew University of Jerusalem, Jerusalem, Israel

**Keywords:** synaptic plasticity, synaptic transmission, neuronal-glial interactions, dendritic spine, neural circuitries formation

Structural and functional specializations of neurons and glial cells, and the dynamical nature of their connections, allow the precise modulation of homeostatic variables and the ongoing behavioral display. Synaptic transmission and plasticity are key cellular processes that enable the nervous system to process information, respond and adapt to a changing environment and internal milieu. In the last few years, the field of synaptic plasticity/modulation has undergone dramatic advances, in particular in the study of the mammalian central nervous system.

This Research Topic provides a state-of-the-art and comprehensive collection of studies focusing on the various mechanisms for modulation of synaptic formation and transmission. Articles depict relevant data about the molecular and structural basis of neuronal cell signaling, plasticity of dendritic spines, synaptic and extrasynaptic transmission, neuronal-glial interactions, modulation of chemical transmitters release, and their functional role, receptor types, and signal transduction up to the level of neural network properties under physiological conditions or related to brain disorders.

Petralia et al. reviewed the structural features to classify invaginating structures at chemical synapses into three categories in the central and peripheral nervous system. Ultrastructural data demonstrate the existence of these elements in presynaptic axonal terminals, postsynaptic spines or dendrites, and glial processes. Spinules and related structures compose the first type of protrusions, which do not present synaptic active zones, whereas the other two categories show active zones within the invagination. Postsynaptic spines that protrude directly into the presynaptic terminal exemplify the second type. Presynaptic terminals that protrude directly into the postsynaptic structure, such as at the neuromuscular junction, represent the third type. Evidence indicate that these specialized invaginating structures have to be carefully considered when further evaluating the mechanisms for neuronal cell signaling.

Mederos et al. reviewed the existing data supporting the crucial roles of astrocytes in synaptic function. These authors described the correlation between structurally- and functionally- different astrocyte populations, by highlighting the current data regarding the heterogeneity of anatomical, molecular, and functional properties of astrocyte–neuron communication. This article critically demonstrates the specialized role of these glial cells in the synaptic transmission and plasticity in different brain areas.

Del-Bel and De-Miguel reviewed the mechanisms of extrasynaptic release of transmitters, by exocytosis or diffusion, from the soma, axon, and dendrites in the absence of postsynaptic counterparts. These authors compared the mechanisms of classic transmitters release, peptides, nitric oxide, and cannabinoids and explored how extrasynaptic transmission interacts to modulate visual sensitivity and blood flow. It is commented on the importance of considering extrasynaptic communication as an important component to understand the function of different neural circuits.

Repetto et al. provided original data into specific roles of individual domains of the multidomain protein Neurobeachin (Nbea) in spine formation, postsynaptic neurotransmitter receptor targeting, and actin distribution. By using live cell imaging and patch-clamp electrophysiology, these authors monitored the structure and function of spinous synapses in primary hippocampal neurons of wild type and Nbea KO mutant mice. They showed the function of specific domains of Nbea in restoring normal dendritic spine density and surface targeting of AMPAR subunits, and in regulating filopodia extension. Interestingly, as the heterozygous mutations in Nbea occur in autistic patients, these findings provides new understanding of the mechanism underlying neuropsychiatric disorders associated with impairments of spine function.

Aubrey and Supplisson provided original data on the heterogeneous signaling at GABA and glycine co-releasing terminals. Using cultured spinal neurons and a combination of loose-patch and whole-cell electrophysiology, these authors have demonstrated that miniature inhibitory postsynaptic currents (mIPSC) originated from terminals containing both GABA and glycine. Their modeling approach predicts that, when glycine gradually replaces GABA in synaptic vesicles, “the redistribution between the peak amplitude and charge transfer of mIPSCs acts to maintain the strength of inhibition while increasing the temporal precision of signaling.” These new data add considerably to the interpretation of changes in synaptic signaling, the strength and plasticity of inhibitory transmission, and the functional role for the corelease of GABA and glycine at central synapses.

Cheng et al. used genetic, electrophysiological, and pharmacological approaches in cultured hippocampal neurons to determine the role of protein kinases and synapsins in two forms of activity dependent, short-term synaptic plasticity that enhance neurotransmitter release. In this original research article, authors provided novel evidence that PKA and synapsins, apparently the main substrates of PKA, are important for augmentation of spontaneous glutamate release at excitatory synapses. These results elucidate important signaling pathways involved in these two forms of short-term plasticity.

Fontes-Dutra et al. showed original results on altered localization of parvalbumin expressing neurons and reduced level of gephyrin in primary sensory cortex in the valproic acid animal model of autism spectrum disorder (ASD). Their results highlighted the importance of changes in GABAergic transmission during brain development. In addition, they demonstrated that resveratrol could have an important effect in rescuing the gephyrin expression in the studied rats.

Rajamani et al. reviewed the role of the neuropeptide oxytocin (OXT) as modulator of synaptic plasticity and neural activity in circuits that regulate social behavior in neurodevelopmental disorders, including ASD. This work highlights studies that report specific alterations in the OXT system in rodent models and explored the potential convergence between the OXT system and genes associated with brain disorders, focusing on the SHANK3 gene. These authors showed evidence supporting the hypothesis that failure of the OXT system during early development could affect social behavior by altering synaptic activity and plasticity.

Rigas et al. report original findings on the impact of early life seizures (ELS) on the network dynamics of the mouse neocortex. Single or multiple seizures were induced using pentylenetetrazole at two different brain developmental stages (postnatal days 9–15 or 19–23), and cortical electrophysiology was assessed by comparing spontaneous network activity (in the form of recurring Up states) in slices of the primary motor and the somatosensory areas of animals at adulthood. Interestingly, results showed that long lasting changes induced by seizures depend on their severity, are region-specific, and are related to the brain maturation period at which the seizures are induced. For example, “single intermittent ELS at P19–23 had no effect on Up state activity, but multiple seizures induced during the same period caused a significant change in the spectral content of spontaneous Up states.”

Quillfeldt provides a Hypothesis and Theory article, discussing a series of findings in which systems consolidation temporal framework changes according to the nature of the behavioral task interposed between the training and the remote test. He proposes a hypothetical “reset” mechanism acting upon a fixed/limited pool of plastic synapses in the CA1 hippocampal area, according to their level of occupancy, to explain the observed temporal flexibility of systems consolidation. The occupancy/reset theory conceives putative CA1 synapse populations with different levels of ability to reset, providing not only a common basis for both synaptic and systems consolidation, but also explaining the different dynamics of episodic and semantic memories.

These current articles highlighted relevant mechanisms of synaptic activity with physiological and/or pathological implications. Indeed, the spatiotemporal modulation of synaptic function is crucial for emergent properties of neural cells, networks, and behaviors. This is a broad and vivid research area. We foresee new approaches and working hypotheses from the data of this Research Topic to the endeavor of understanding the functional organization of the central nervous system.

## Author Contributions

All authors listed have made a substantial, direct and intellectual contribution to the work, and approved it for publication.

### Conflict of Interest Statement

The authors declare that the research was conducted in the absence of any commercial or financial relationships that could be construed as a potential conflict of interest.

